# Characterization of the cytokinin sensor *TCSv2* in arabidopsis and tomato

**DOI:** 10.1186/s13007-020-00694-2

**Published:** 2020-11-16

**Authors:** Evyatar Steiner, Alon Israeli, Rupali Gupta, Ido Shwartz, Ido Nir, Meirav Leibman-Markus, Lior Tal, Mika Farber, Ziva Amsalem, Naomi Ori, Bruno Müller, Maya Bar

**Affiliations:** 1 Institute of Plant Sciences and Genetics in Agriculture, Hebrew University of Jerusalem, 7610001 Rehovot, Israel; 2 Department of Plant Pathology and Weed Research, Plant Protection Institute, Agricultural Research Organization, The Volcani Center, 7505101 Rishon LeZion, Israel; 3 Department of Plant and Environmental Science, Weizmann Institute of Science, 7610001 Rehovot, Israel; 4 Leibniz-Institut Für Pflanzengenetik Und Kulturpflanzenforschung (IPK), Corrensstraße 3, 06466 Seeland, Germany; 5 Department of Biology, Stanford University, Stanford, CA 94305 USA; 6 Department of Plant Biology, University of California – Davis, Davis, CA 95616 USA; 7 Microsynth AG, Schützenstrasse 15, 9436 Balgach, Switzerland

**Keywords:** Cytokinin, Imaging, Two-component sensor (*TCS*), *TCSv2*, Tomato, Arabidopsis

## Abstract

**Background:**

Hormones are crucial to plant life and development. Being able to follow the plants hormonal response to various stimuli and throughout developmental processes is an important and increasingly widespread tool. The phytohormone cytokinin (CK) has crucial roles in the regulation of plant growth and development.

**Results:**

Here we describe a version of the CK sensor *Two Component signaling Sensor* (*TCS*), referred to as *TCSv2*. *TCSv2* has a different arrangement of binding motifs when compared to previous *TCS* versions, resulting in increased sensitivity in some examined tissues. Here, we examine the CK responsiveness and distribution pattern of *TCSv2* in arabidopsis and tomato.

**Conclusions:**

The increased sensitivity and reported expression pattern of *TCSv2* make it an ideal *TCS* version to study CK response in particular hosts, such as tomato, and particular tissues, such as leaves and flowers.

## Background

Cytokinins (CKs) are a class of adenine-derived plant hormones that control multiple processes throughout the plant life cycle. They provide positional information for growth and patterning, and integrate biotic and abiotic cues from the environment. Notable examples are meristem maintenance in both the shoot apical meristem (SAM) and root apical meristem (RAM), cell division and cell differentiation. CK is also involved in regulating traits that affect yield and fruit quality. The roles of CK in plant growth and development have been reviewed extensively [1–6]. Cytokinin signalling is mediated via a two-component multistep phosphorelay cascade. As the final step, type-B response regulators (RRs) activate transcription in response to phosphorelay signalling activity, while type-A response regulators are rapidly induced by CK via Type-B RRs, and, in turn, repress signalling via a negative-feedback loop [6–10].

There are many CK derivatives, and methods for the detection of a large number of them have emerged in recent years [11–20]. However, it is often difficult to know for certain which of these derivatives represent active CKs, and not all of them are detectable. In parallel to the advances made in hormone substance detection, efforts have also been invested in the detection of CK signalling via transcriptional sensors that mark the site of CK-derived response within a specific tissue or organ. Whereas specific genes and promoters involved in the CK pathway served as markers for CK response in the past [7, 8, 10, 21, 22], limitations in the ability to detect cytokinins and decipher the biosynthetic pathways culminating in active variants of CK molecules led to the necessity for accurate and robust sensors allowing us to follow CK response dynamics *in planta*. The search for a robust and sensitive CK sensor led to the creation of the TWO-COMPONENT OUTPUT SENSOR, *TCS*, which was designed based on the CK phosphorelay network. The *TCS* sensor was designed using the conserved DNA binding domain in the promoter of type A RRs that is recognized by type-B response regulator family members of arabidopsis. With the goal of designing a universal CK reporter, different synthetic reporter designs were optimized using luciferase (LUC) activity in an arabidopsis mesophyll protoplast assay system [7, 8, 23]. The first generation *TCS*, *TCS::LUC*, harboured concatemerized type-B arabidopsis response regulator (ARR) binding motifs and a minimal 35S promoter [7, 8]. The second version of *TCS*, named *TCSn*, was optimized to better reflect the natural arrangement of binding motifs [24]. To minimize transcriptional silencing triggered by repeats, sequence variations in non-relevant residues were introduced in *TCSn*. Since its introduction, *TCSn*-based reporters have proven essential tools to report cytokinin responses in different plant species, including monocots [25, 26].

*TCS* and *TCSn* design were based on analysis of binding motifs in verified cytokinin targets. This analysis revealed that tandem, head-to-head and tail-to-tail motif orientations are all equally frequent [24]. Therefore, both *TCS* and *TCSn* reporters were designed to harbour a motif arrangement that provides all these relative orientations. However, in cellular assays, increased sensitivity was detected in head-to-head and tail-to-tail motif orientations. This led to the design of another *TCS* version, which is described here. The corresponding synthetic promoter, named *TCSv2* (version2) shows increased cytokinin sensitivity *in planta*, in particular in the shoot meristem, making it an ideal choice for detecting CK response in shoot organs. Here, we report the *TCSv2::NLS-3XVenus* expression pattern in transgenic tomato and arabidopsis.

## Results

### TCSv2 design and increased sensitivity

**Fig. 1:**
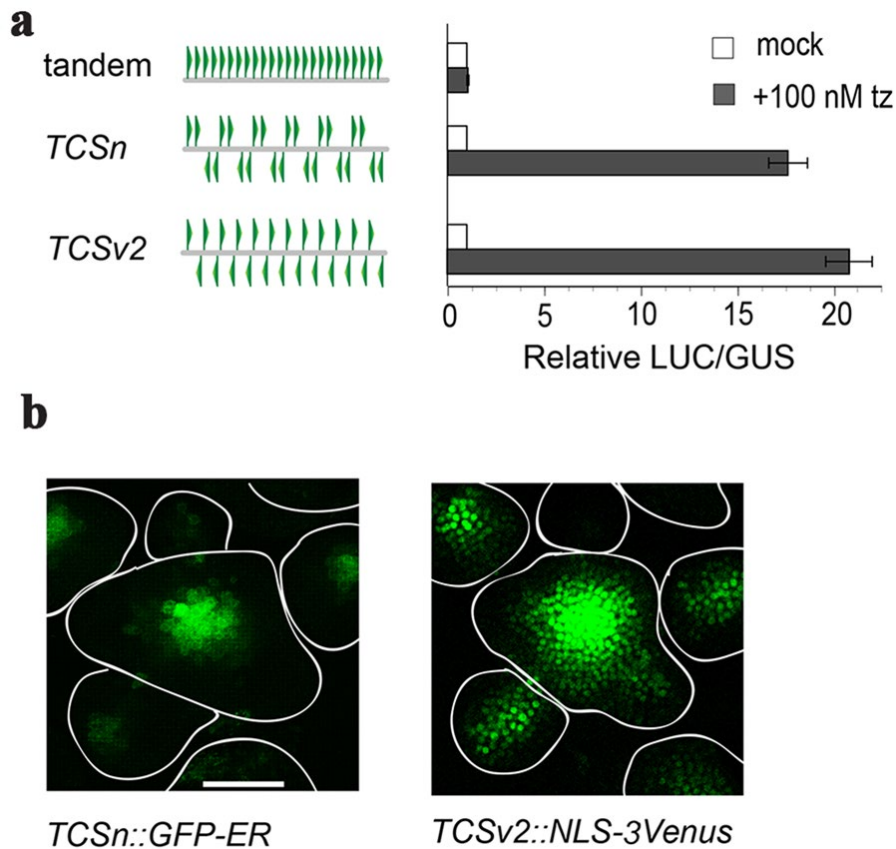
*TCSv2* possesses increased sensitivity. **a** Design scheme of *TCSn* and *TSCv2*, displaying the arrangement of the motifs in the synthetic promoter, alongside activity of the construct (Luciferease/GUS staining) in mock and 100 nM transZeatin (tz) treated samples. **b** Expression of *TCSn* and *TCSv2* driven VENUS in the floral meristem of arabidopsis. Bar = 50 µM

*TCSv2* is a variant of *TCSn*, with alternating head-to-head and tail-to-tail orientations of type B ARR-binding sites compared with the tandem tail-to-tail and head-to-head orientation of sites in *TCSn* (Fig. 1a). *TCSv2* demonstrated increased sensitivity in mesophyll protoplast transient assays (Fig. 1a), as well as in arabidopsis floral meristems (Fig. 1b).

### CK responsiveness of TCSv2 in various tissues in both arabidopsis and tomato

**Fig. 2:**
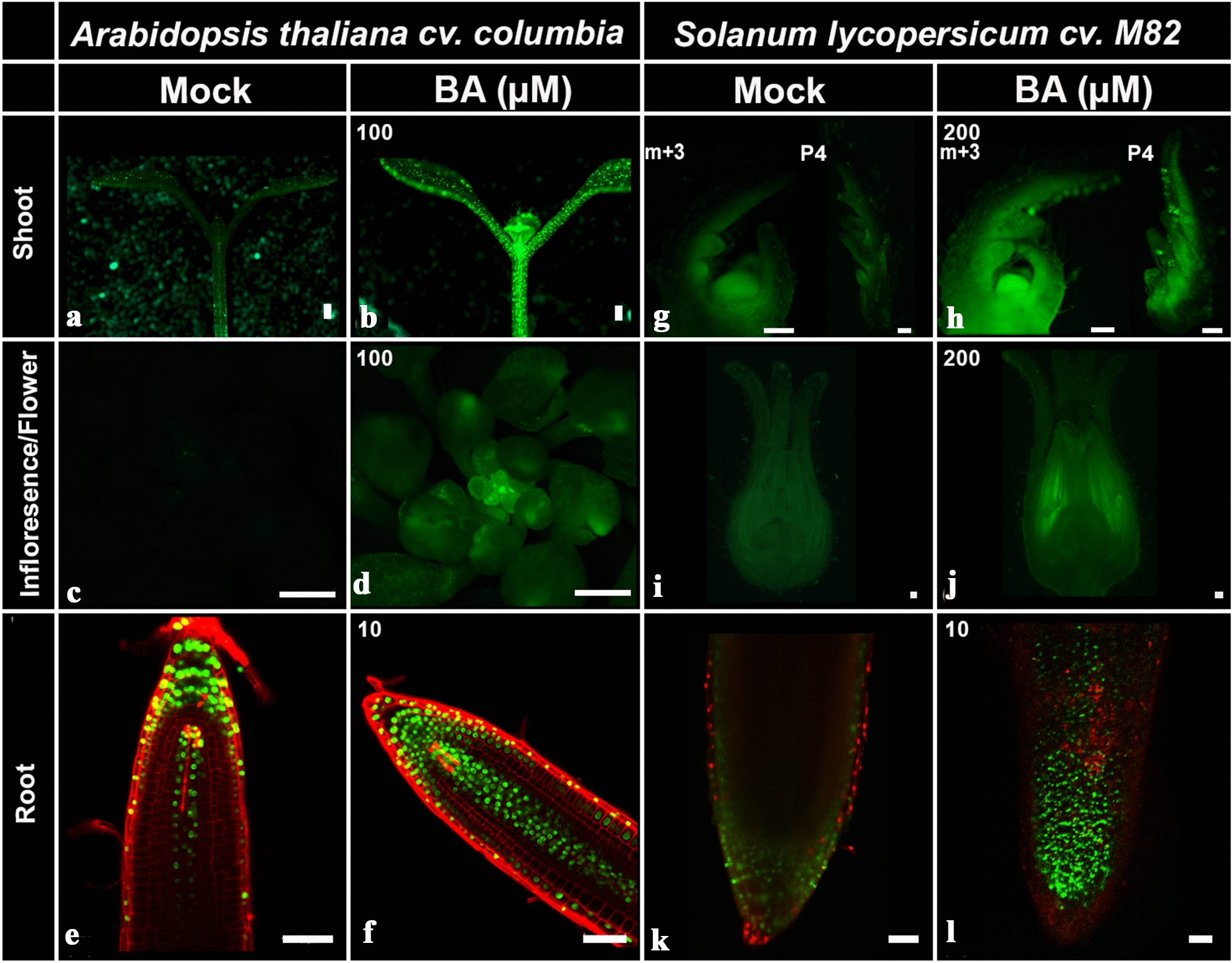
*TCSv2*:3XVENUS responds to CK treatment. Arabidopsis (*A. thaliana* Columbia*)* seedlings (**a**, **b**), inflorescences (**c**, **d**), and roots (**e**, **f**), and Tomato (*S. lycopersicum* M82) apexes (**g**, **h**, developmental stages indicated), flower primordia (**i**, **j**) and roots (**k**, **l**) were treated with mock or indicated 6-benzylaminopurine (BA) concentrations. Images of *TCSv2* driven VENUS expression were captured 24 h after treatment with a Nikon stereomicroscope (**a**–**d**, **g**, **h**), a Leica SPX confocal microscope (**e**, **f**) or a lsm510-META confocal microscope (K-L). Parameters and settings are described in the materials and methods section. Bars = 100 µM

We cloned *TCSv2* according to the description in the methods section, and introduced constructs in which *TCSv2* drives VENUS or GUS (β-glucuronidase) expression into tomato and arabidopsis. To examine CK responsiveness of *TCSv2 *in vivo, we conducted a series of experiments examining *TCSv2* driven expression with and without CK treatment in a variety of plant tissues in both arabidopsis and tomato (Fig. 2). For VENUS analyses conducted in arabidopsis, two representative transgenic lines exhibiting moderate (*TCSv2*:3XVENUS#2) and strong (*TCSv2*:3XVENUS#7) VENUS expression were selected for the analysis. For VENUS analyses conducted in tomato, several lines were screened, which demonstrated similar VENUS expression levels. One of these was selected for further analyses.

*TCS* driven VENUS expression is observed primarily in meristematic tissues in the arabidopsis shoot and root, as was reported for previous *TCS* versions (Fig. 2a, e). Similarly, strong expression was observed in tomato SAM and RAM (Fig. 2g, k). Interestingly, in tomato, strong expression was also observed in the leaf marginal blastozone (Fig. 2g), a meristematic region present in leaf margins and expanded in margin of compound leaves [27]. Upon CK treatment, the VENUS pattern of expression expands to the cotyledons and hypocotyl in arabidopsis (Fig. 2b), and becomes stronger in tomato shoot apexes (Fig. 2h). In roots of both arabidopsis and tomato, *TCS* driven VENUS expression is observed in the root apex, presumably localized to the root apical meristem, as well as in the columella and internal stele (Fig. 2e, k). This pattern is strengthened and expanded following CK treatment (Fig. 2f, l). *TCSv2*:NLS-3XVENUS may respond to CK treatment in a dose dependent manner (Additional file 1: Figure S1). Results obtained with *TCSv2* in a *Ler* arabidopsis background were similar (Additional file 1: Figure S2). For some tissues, such as shoot apices, tomato may require a higher concentration of CK to achieve a similar strength response in the same time frame as arabidopsis.

**Fig. 3:**
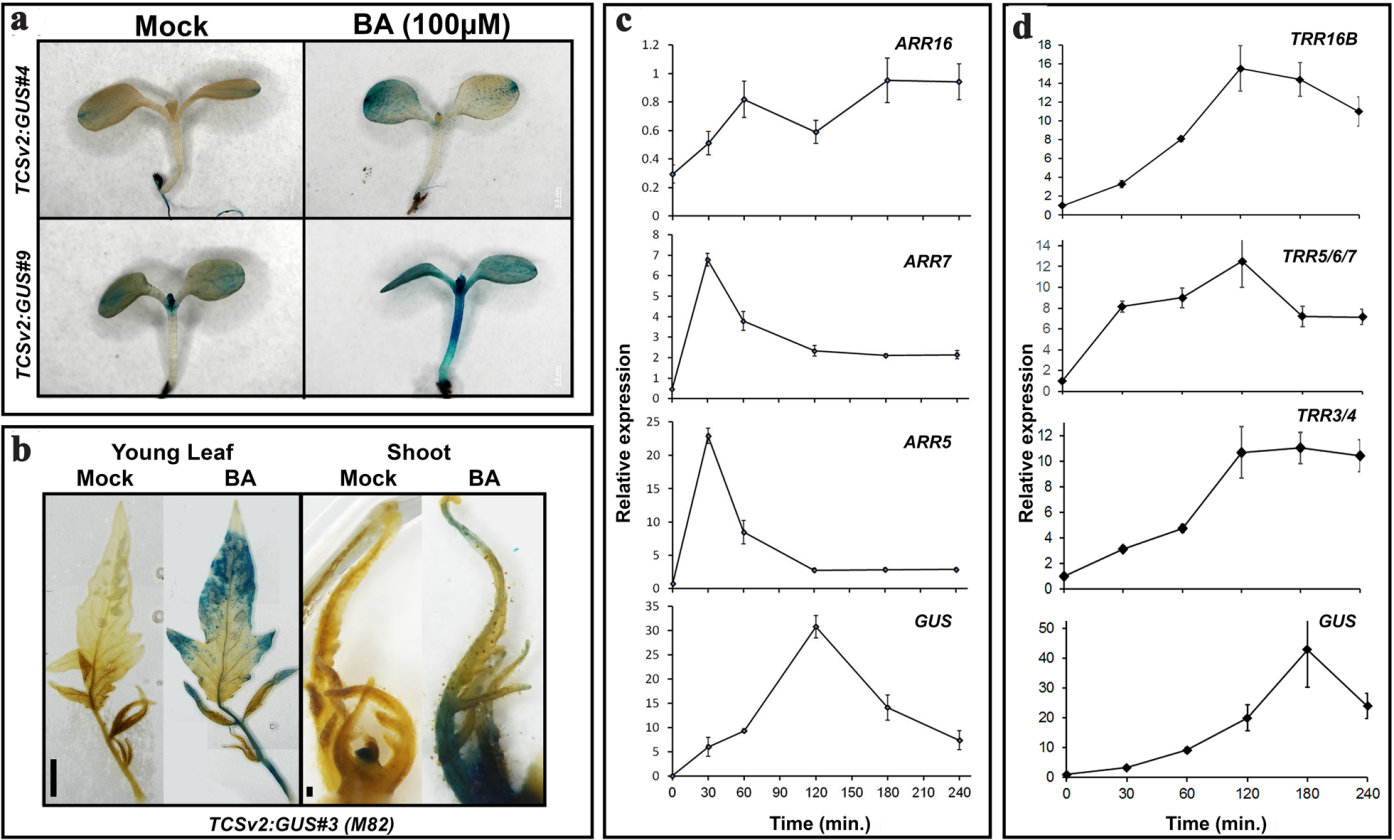
*TCSv2* time course following CK treatment. Arabidopsis (*A. thaliana* Columbia*)* seedlings (**a**), and Tomato (*S. lycopersicum* M82) leaves and apexes (**b**) were assayed for GUS accumulation with and without CK treatment (6-benzylaminopurine, BA; 100 µM) tissues were harvested 2 and 4 h after treatment respectively). Images were captured with a Nikon stereomicroscope. Bars = 1 cm. *GUS* and response regulator *ARR/TRR* relative expression were assayed at indicated time points after CK treatment in arabidopsis (**c**) and tomato (**d**). Each point represents at least 3 biological replicas ± SE

In order to examine the time course of the response of *TCSv2* to CK treatment, we utilized a *TCSv2*:GUS construct (the VENUS construct contains 3 repeats of the VENUS sequence and is therefore unsuitable for qPCR analysis). We first characterized GUS expression in arabidopsis and tomato (Fig. 3). Time course experiments show that *TCS*-driven *GUS* mRNA peaks 2 h after CK treatment in arabidopsis (Fig. 3c), and declines soon thereafter. Examination of *TypeA ARR* genes in the same samples, shows that, as previously reported [28, 29], *ARR5* and *ARR7* respond earliest, within half an hour of CK treatment (Fig. 3c). In tomato, *TCS*-driven *GUS* mRNA peaks 3 h after CK treatment (Fig. 3d), rising and falling slightly slower than in arabidopsis, perhaps reflecting the higher amount of CK needed to elicit a similar response. When comparing *TRR* expression in tomato to that of the *TCS-GUS* mRNA, *GUS* mRNA expression rises in a manner similar to that of *TRR16B* and *TRR3/4*, but to a greater degree, rising slowly and peaking 2–3 h from CK treatment, while *TRR5/6/7* rises more quickly, showing significant increase in expression 30 min after CK treatment, similar to arabidopsis *ARR7* (Fig. 3c, d). *TCSv2* can thus be viewed as an “averaging” output of *Type A ARR* response in terms of time course, representing a response to CK that is later than the earliest responding *ARRs* but earlier than the later responding ones. Also of note is that in both arabidopsis and tomato, *TCSv2* responds more strongly to CK treatment than any one individual RR, perhaps reflecting a combined response output that is normally “divided” between several RR genes. This should be taken into account when conducting analyses using *TCSv2*.

### TCSv2 driven expression is affected by alterations in endogenous CK levels in tomato

**Fig. 4:**
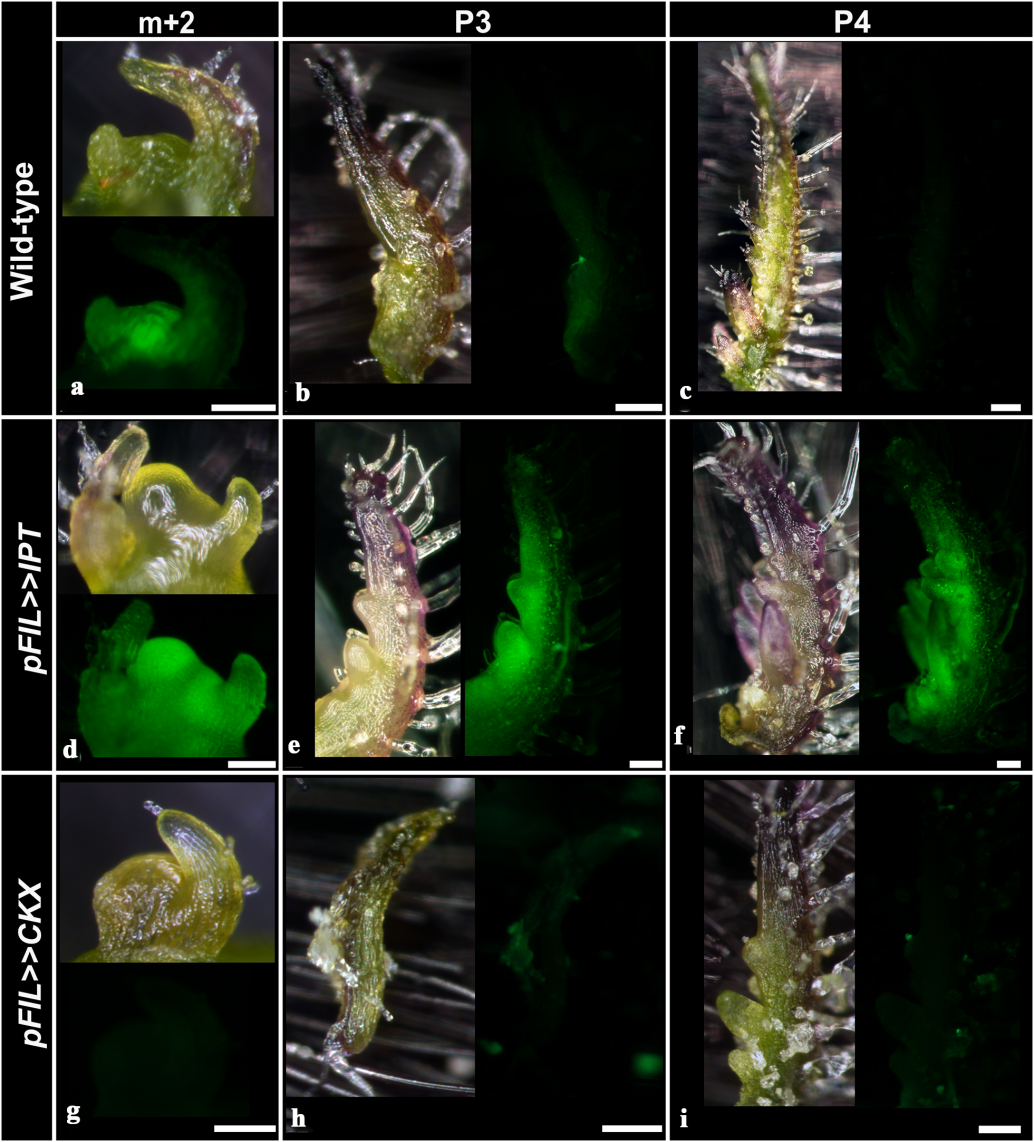
Endogenous CK alterations affect *TCSv2* driven expression. Characterization of *TCS* driven VENUS expression in wild type (WT) Tomato shoot apexes and leaf primordia (*S. lycopersicum* M82) (**a**–**c**), in comparison with apexes and primordia of tomato plants overexpressing *pFIL* >  > *IPT7* (**d**–**f**) and *pFIL* >  > *CKX3* (**g**–**i**). Images were captured with a Nikon stereomicroscope. Bars = 100 µM

To examine whether *TCSv2*-driven expression responds to alterations in endogenous CK level alterations in tomato, we backcrossed the tomato VENUS line into transgenic plants overexpressing the arabidopsis CK biosynthesis enzyme Isopentenyltransferase-7 (IPT7) or CK catabolic enzyme Cytokinin Oxidase/Dehydrogenase -3 (CKX3) [30], driven by the *FIL* promoter. As can be seen in Fig. 4, the *TCSv2* sensor responds to an increase in endogenous CK with elevation of VENUS expression (Fig. 4d–f) and to a decrease in endogenous CK with a decrease in VENUS expression (Fig. 4g–i), indicating that the sensor is useful for examining both exogenous and endogenous changes in CK levels. Indeed, we recently successfully utilized the *TCSv2* sensor to analyse endogenous effects of different genetic background manipulations on the CK pathway [31–33].

### TCSv2 responds primarily to CK treatment

**Fig. 5:**
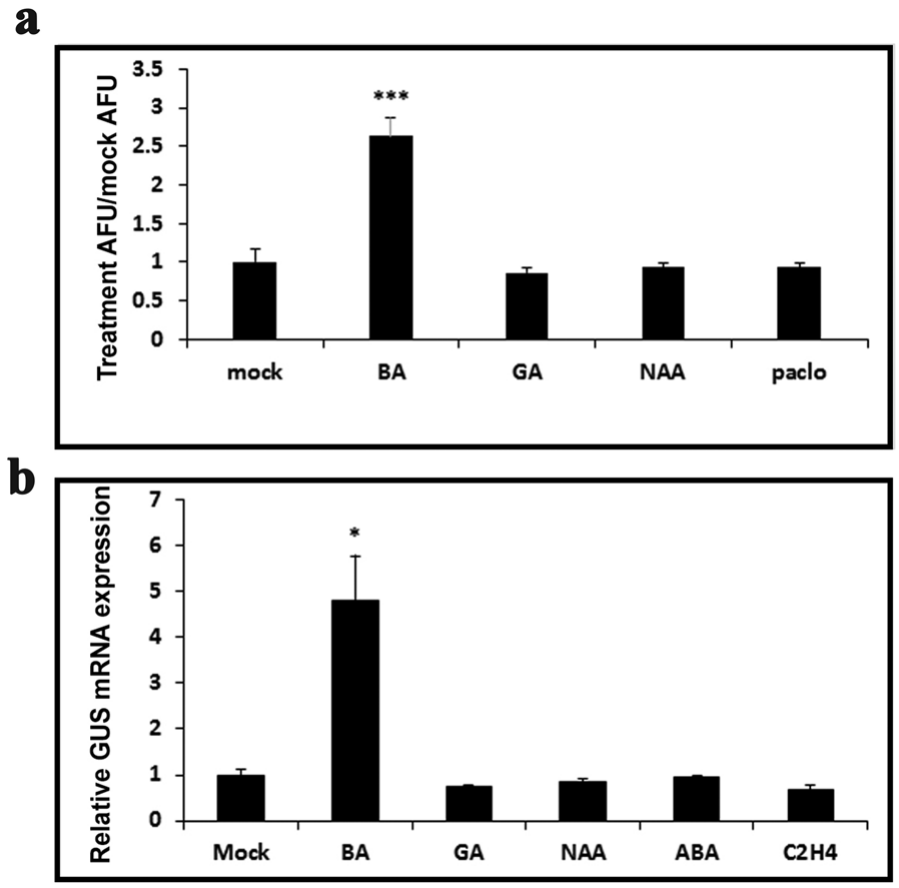
*TCSv2* responds primarily to CK. **a** Quantification of *TCS* driven VENUS expression in wild type Tomato shoot apexes following treatment with indicated hormones. VENUS expression was quantified as arbitrary fluorescent units (AFU) using ImageJ software [62], using images captured with a Nikon stereomicroscope (5–8 shoots per sample). Representative images are shown in Additional file 1: Figure S3. **b** *GUS* relative expression was assayed 2 h after treatment with indicated hormones in arabidopsis. Each point represents at least 3 biological replicas ± SE. In both **a** and **b**, Student’s t-test (two-tailed) was used for comparison of means, which were deemed significantly different at P ≤ 0.05

The balance between different hormones is one of the underlying mechanisms serving plant development, growth, and response to various cues. Cytokinin and gibberellin, as well as cytokinin and auxin, can antagonize each other or act in concert in a variety of processes throughout plant development [30, 34–43]. We therefore tested whether the *TCS* sensor could possibly respond to additional cues other than CK treatment. Figure 5 demonstrates that *TCSv2* responds specifically to CK and does not respond significantly to additional tested hormones at the applied concentrations and within the indicated time frames, in both tomato (VENUS protein expression 12 h after CK treatment, Fig. 5a and Additional file 1: Figure S3A–E) and arabidopsis (*GUS* mRNA expression 2 h after CK treatment, Fig. 5b). This indicates that the *TCSv2* sensor is specific and accurate, in addition to being robust, in the detection of CK response in plants.

### Characterization of TCSv2 driven expression throughout development

**Fig. 6:**
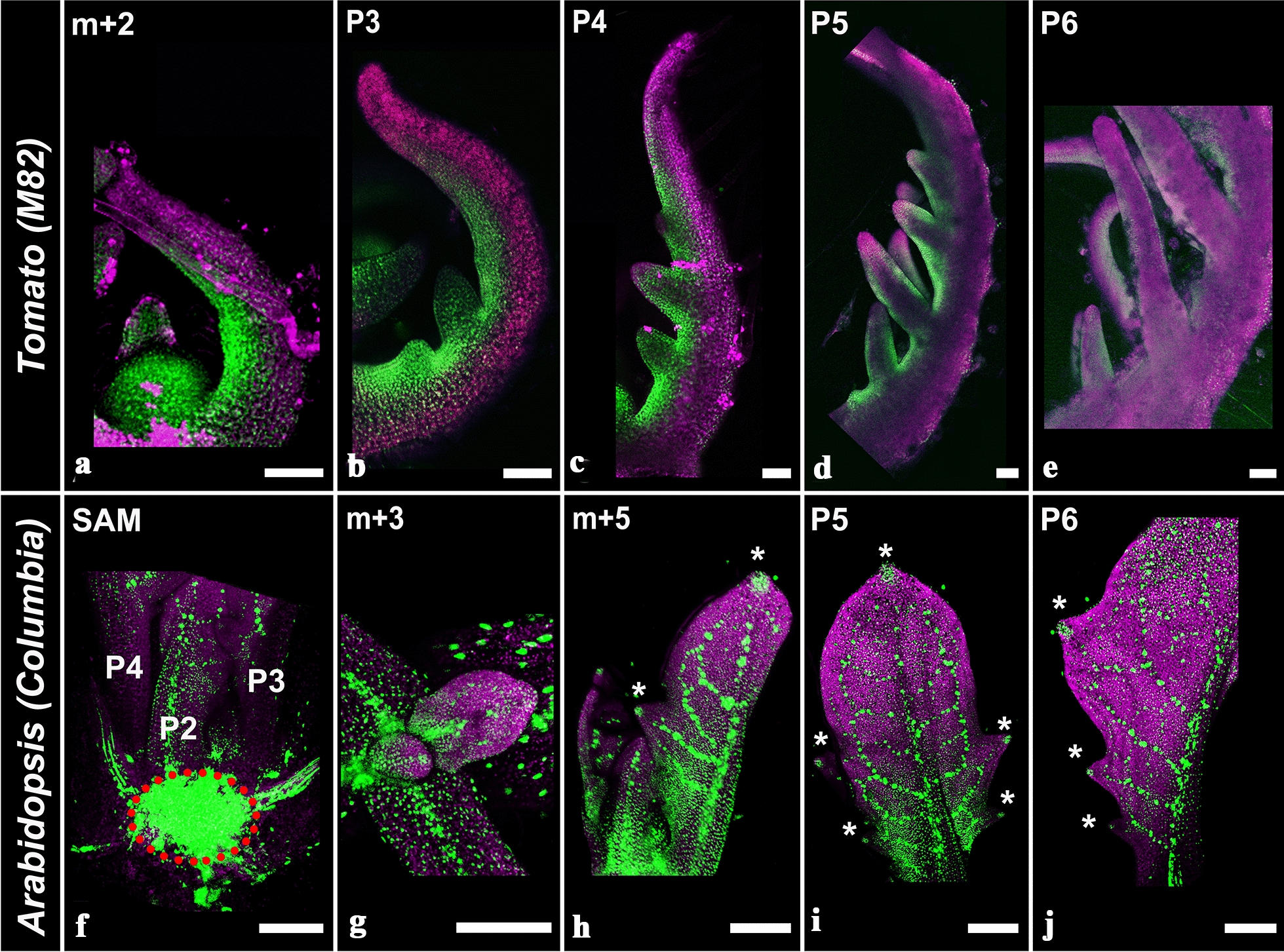
*TCSv2* driven expression during leaf development. *TCSv2* driven expression at various stages of leaf development in tomato (**a**–**e**) and arabidopsis (**f**–**j**). The dotted red circle in F marks the shoot apical meristem. Images were taken with a lsm780 confocal microscope. Bars = 100 µM

The observation that *TCSv2* is primarily visible in meristematic tissues, along with published analyses of previous *TCS* versions in arabidopsis development, prompted a more in depth examination of *TCSv2* in various developmental contexts. Figure 6 presents an analysis of *TCSv2* driven expression throughout shoot and leaf development in tomato (Fig. 6a–e) and arabidopsis (Fig. 6f–j). In tomato, *TCSv2* is expressed in the SAM and at the margin of young leaf primordia. The *TCS* expression domain, which likely correlates with the marginal blastozone [27], appears to be wider in younger leaf primordia (Fig. 6a–c), and becomes localized and quite thin in older leaf primordia (Fig. 6d, e), consistent with the notion that the young leaf undergoes morphogenesis and reaches maturation concurrently with the loss of its morphogenetic potential and meristematic tissues. This is evident in Additional file 1: Figure S4, which shows older tomato leaves in which the *TCS* driven signal is localized to the margins of the developing leaflets only (Additional file 1: Figure S4E, the VENUS signal was color coded in dark blue to make it more visible). In contrast, in arabidopsis, which has a simple leaf, the limited morphogenetic potential retained by young leaf primordia serves in the execution of leaf marginal patterning. As such, *TCSv2* driven expression can be observed in the SAM (Fig. 6f), and at the adaxial side of the leaf base in young leaf primordia (P1–P3, Fig. 6g). The *TCSv2* driven signal is restricted as the leaf matures, in-line with the basipetal differentiation of the arabidopsis leaf. *TCSv2* is also present throughout the young leaf venation (Fig. 6h–j), and localized to a small number of cells which mark the leaf tip and the peak/tip of a nascent marginal serration (Fig. 6h–j, marked with asterisks), which presumably maintain some form of meristematic qualities to allow for subsequent leaf marginal patterning, which is dependent on CK response. Consistent with the short marginal blastozone activity, *TCSv2* is not observed throughout the leaf margin in arabidopsis*.*

### TCSv2 marks the zone of the incipient axillary bud

**Fig. 7:**
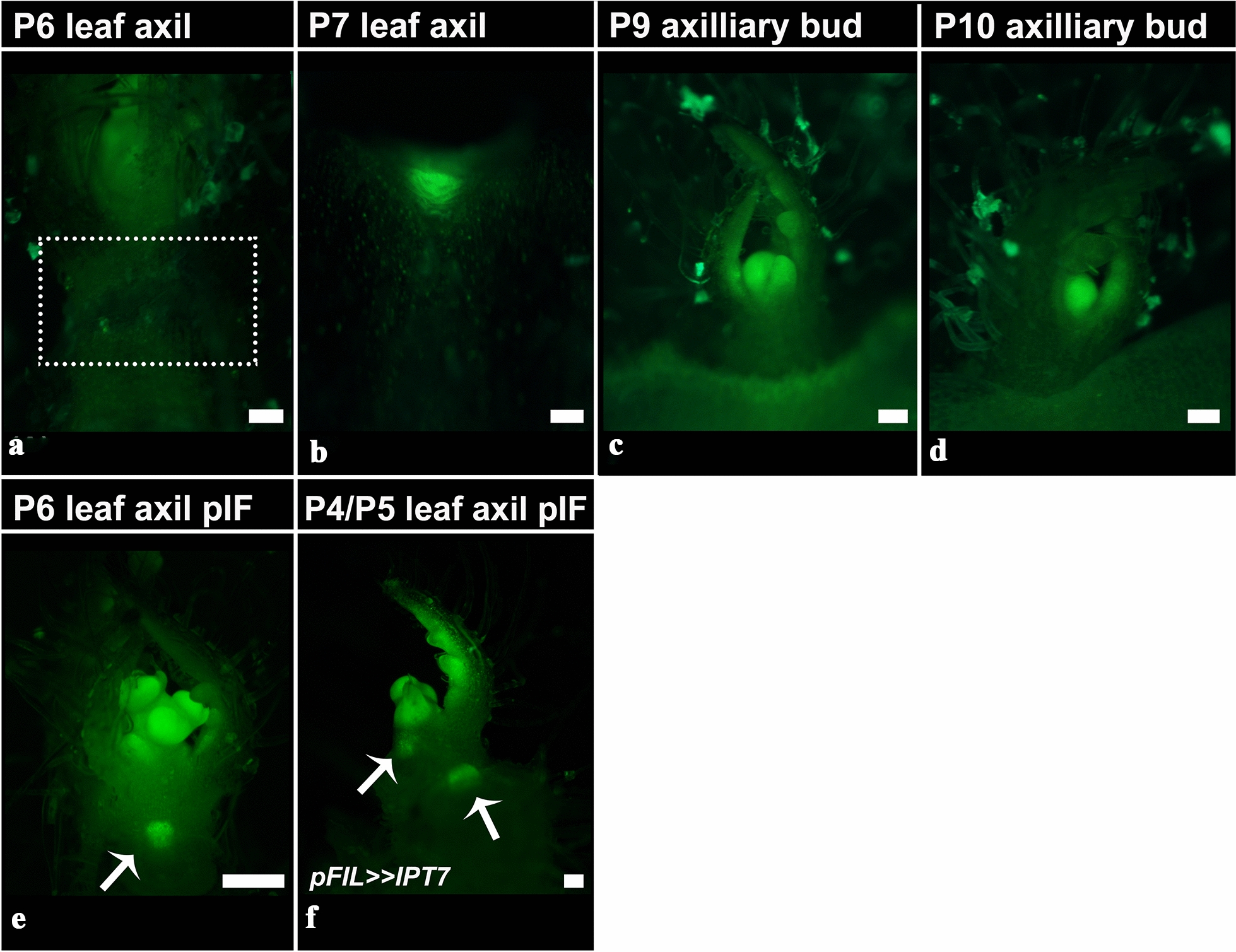
*TCSv2* driven expression during axillary bud/shoot activation in tomato. *TCSv2* driven expression in tomato leaf axils of leaves at different developmental stages, before (**a**–**d**) and after (**e**, **f**) flowering induction. Dotted box in A marks the axil of the removed P6. Arrows in **e** and **f** indicate the axils of leaves that were removed. Images were taken with a Nikon stereomicroscope. Bars = 100 µM

CK response has been reported to be crucial in the establishment of the axillary bud [44, 45]. Utilizing *TCSv2*, we followed the generation of the axillary shoot in tomato. We were able to observe CK response signal in the axils of leaf primordia from the P7 stage onward (Fig. 7). At P7, the *TCSv2* signal is present in the leaf axil, though no axillary bud or axillary meristem dome has yet formed (Fig. 7b). At later stages, in the P8-P10 axil, an activated bud with a characteristic *TCSv2* signal in the meristem and the margins of the developing leaf primordia can be observed (Fig. 7c, d). Interestingly, after induction of flowering, we see *TCSv2*-driven expression in the axils of younger, P6 primordia (Fig. 7e). When the reproductive transition state is coupled with elevation of endogenous Cytokinin present in *pFIL* >  > *IPT7* overexpressing plants, the *TCSv2* signal is observed in the axils of even younger primordia—P4 and P5 (Fig. 7f). Notably, *TCS* driven GFP expression using the first *TCS* version [8] was observed in the leaf axils of P6 and older leaf primordia in arabidopsis [46].

### TCSv2 driven expression is observed in all stages of reproductive organ development

**Fig. 8:**
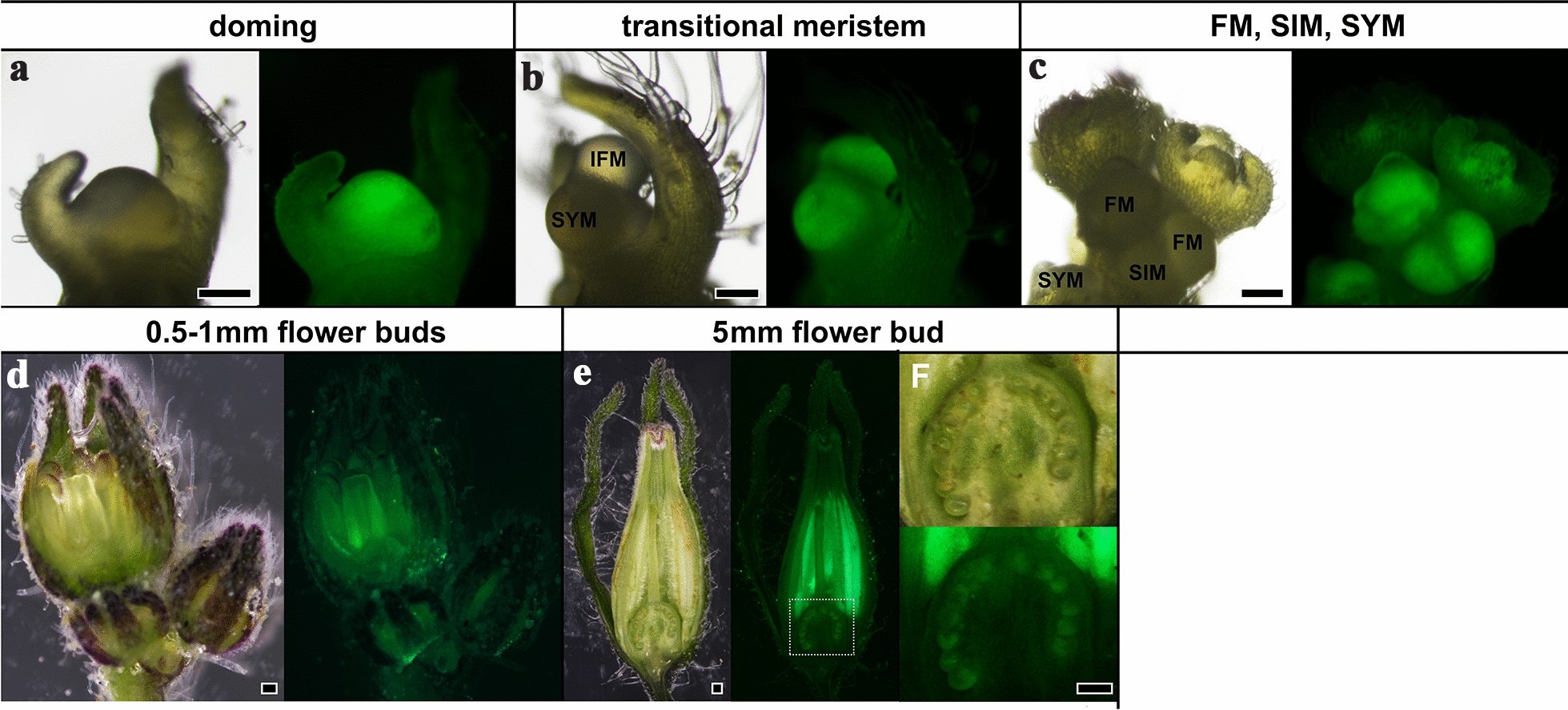
*TCSv2* driven expression during flower development in tomato. *TCSv2* driven expression at various stages of flower development in tomato. Images were taken with a Nikon stereomicroscope. *FM* floral meristem, *IFM* inflorescence meristem, *SIM* sympodial inflorescence meristem, *SYM* sympodial meristem. Bars = 100 µM

As reported previously and here above, CK response and *TCSv2* driven expression are primarily observed in meristematic tissues. This is the case also in reproductive organ development (Fig. 8). *TCSv2* is observed during the transition to flowering in the domed meristem (Fig. 8a), the transitional meristem (Fig. 8b), and the inflorescence and floral meristems (Fig. 8c). Once reproductive organs have formed, we can observe *TCSv2* in the anthers and filaments (Fig. 8d, e) and the ovules (Fig. 8e), indicating that CK response is required for proper reproductive organ development.

In mature embryos, *TCSv2* driven expression is observed very strongly in the region of the RAM (Additional file 1: Figure S5A–C), but, interestingly, can barely be seen in the progenitor cells of the SAM, both in desiccated and imbibed seeds, even after germination (Additional file 1: Figure S5A–C). Following cotyledon expansion and generation of the nascent SAM structure, *TCSv2* driven expression can be observed in the meristem and young P1 (L2) and P2 (L1) leaves (Additional file 1: Figure S5D, E). Different versions of *TCS* can therefore be used in order to obtain a full picture of CK response in plant tissues. A combination of different *TCS* promoters could be useful, depending on the exact nature of the processes being observed.

## Discussion

Here we report a new version of the CK sensor TCS, which is more sensitive than previous versions, and characterize its expression patterns in response to CK treatment and during developmental processes in both arabidopsis and tomato. The differences between the *TCS* versions should be taken into account when considering which version of *TCS* to work with.

During leaf development, the young leaf undergoes morphogenesis and reaches the maturation stage of its development concurrently with the loss of its morphogenetic potential. The morphogenetic potential of tomato leaves serves to model compound leaves bearing leaflets, from a meristematic region termed the marginal blastozone [27], while in arabidopsis the more limited morphogenetic potential of young leaf primordia serves in the execution of leaf marginal patterning, resulting in serrations at the leaf margin. The leaf morphogenetic potential is marked by meristematic / totipotent / stem/ cells, which respond to CK and exhibit *TCS* driven expression. The tomato compound leaf retains morphogenetic potential, and expresses *TCSv2* throughout the leaf margin. The older the leaf becomes, the lower its morphogenetic potential, and thus, the weaker and more localized the *TCS* signal becomes. *TCSv2*, and likely other *TCS* versions, could therefore serve as a marginal blastozone marker in tomato. In arabidopsis, *TCSv2* driven expression is absent from the leaf margins, but is retained in the leaf base, where undifferentiated cells remain for a longer time during development. Leaf differentiation is a gradual process, and in many plant species, cell differentiation and expansion progress from the leaf tip towards the base in a moving “cell cycle arrest front” [47, 48].

*TCSv2* also marks CK response in axillary bud formation and activation in tomato. CK is known to be required for axillary bud activation [49]. CK biosynthesis was suggested to correlate with bud outgrowth [44], and auxin was demonstrated to regulate the synthesis of CKs [50] and negatively regulate local biosynthesis of CKs by controlling the expression of isopentenyltransferase (IPT) genes [51], suggesting that auxin-dependent apical dominance is exerted, at least in part, by inhibiting axillary bud growth via CK inhibition. However, this is likely more complex, as recently it was reported that defects in bud CK response do not affect auxin-mediated bud inhibition in arabidopsis [52]. Observing *TCSv2* driven expression in tomato leaf axils, we were able to determine that axil CK response is activated in developmentally younger leaf axils after the plant has undergone an induction to flowering, perhaps suggesting that the apical dominance of the plant is somewhat reduced after it has transitioned to its reproductive stage. Indeed, it was recently demonstrated that increased CK levels in subapical axillary buds coincide with a release from apical dominance after floral transition in chrysanthemum [49]. We also showed that apical dominance is reduced further when the CK pathway is manipulated, consistent with published data that overproduction of CK in leaf axils can rescue axillary meristem initiation deficiency in *rax* mutants in arabidopsis [53], and that CK application to tomato seedlings promotes axillary bud outgrowth [54].

*TCSv2* driven expression was observed in ovules and stamens, demonstrating that CK response is required for proper reproductive organ development during the reproductive stage in tomato. Indeed, mutants impaired in the CK pathway have varying degrees of reduced fertility [55–58]. It will be interesting to examine *TCSv2* driven expression during the various stages of flower and fruit development in tomato.

## Conclusions

We present here a new version of the *TCS* CK sensor, *TCSv2*, which responds to CK in various tissues in both arabidopsis and tomato. *TCSv2* proved very useful in following several developmental processes and is an important addition to those interested in following CK responses *in planta*, with the exception of embryo development.

## Materials and methods

### Cloning and plant transformation

The CK responsive promoter-reporter TWO-COMPONENT OUTPUT SENSOR VERSION2 (*TCSv2*) harbours concatamerized type B ARR-binding motifs and is a variant of *TCSn* [24], with alternating head-to-head and tail-to-tail orientations of type B ARR-binding sites compared with the tandem tail-to-tail and head-to-head orientation of sites in *TCSn*. *TCSv2* reflects the activity of the CK phosphorelay cascade. The sequence of *TCSv2* is 5′-CAAAGATTTTGCAAAATCTTTTAAAGGATTTTGAAAGATCTTTGCAAAGATCTTTATAAATCTTTTCAAAGATTTTTCAAGATCCGATTAAAGATTTTGCAAAATCTTTAGAGAGATCTTTCAAAATCCAACGCTAGTCAAAGATTTTGCAAAATCTTTTAAAGGATTTTGAAAGATCTTTGCAAAGATCTTTATAAATCTTTTCAAAGATTTTTCAAGATCCGATTAAAGATTTTGCAAAATCTTTAGAGAGATCTTTCAAAATCCAAC-3′.

For *TCSv2*:3XVENUS, the DNA sequence of *TCSv2* was synthesized with flanking NsiI and BamHI restriction sites [32]. The synthetic promoter was then cloned adjacent to 3xVENUS-N7 in the pBJ36 vector [59]. The construct was subcloned into the pGREEN binary vector. For *TCSv2::GUS*, *TCSv2* was ligated to the β-galactosidase (GUS) gene from *Escherichia coli* to generate a *TCSv2:GUS* fusion in pART27 [33]. Constructs were introduced into arabidopsis *Ler* and *Col* backgrounds by floral dipping, and into tomato M82 according to [60]. Kanamycin resistant transformants were selected.

Transgenic tomato plants overexpressing *pFIL* >  > *IPT7* and *pFIL* >  > *CKX3* have been described previously [30].

### Transient protoplast expression

Protoplast isolation and transfection experiments were performed as reported [23, 24]. All protoplast experiments were performed in duplicates, and independent biological replicates yielded similar results.

### CK and hormone treatments

All exogenous applications of the synthetic CK 6-benzylaminopurine (BA) (Sigma-Aldrich, St Louis, MO, USA) were performed by spraying or immersing the plants for 5 min. Absicic acid (ABA, 100 µM), 1-Naphthaleneacetic acid (NAA, 100 µM), gibberellic acid (GA, 100 µM) paclobutrazol (paclo, 10 mg/mL), all from Sigma-Aldrich, and Ethylene (Ethrel, Bayer Cropscience) were applied by spraying. All hormone treatments included the surfactant Tween 20 (100 μl l^−1^).

### Tissue preparation and imaging

Dissected whole-leaf primordia, shoots, leaves, leaf axils, inflorescences, flowers, roots and embryos were placed into drops of water on glass microscope slides and covered with cover slips. Roots and embryos were stained with PI (Propidium iodide, Sigma-Aldrich P4170, 10-20ug/mL final concentration in water for 2 min with subsequent washing) prior to mounting. GUS staining was carried out essentially as described in Ori et al., 2000 [61]: Plant tissue was vacuum infiltrated for 1 min in a solution containing 25 mM phosphate buffer, pH 7, 0.25% Triton X-100, 1.25 mM potassium ferricyanide, 1.25 mM potassium ferrocyanide, 0.25 mM EDTA, 1 mg/ml 5 bromo-4 chloro-3-indolyl-β-D-glucuronide (X-Glucoside, Inalco Pharmaceuticals), and incubated overnight at 37 °C. Tissue was then cleared in 95% ethanol, gradually brought to 50% ethanol and then to 50% glycerol. Tissue was photographed in 50% glycerol.

The pattern of VENUS expression was detected by a confocal laser scanning microscope (CLSMmodel SP8; Leica), with the solid-state laser set at 514 nm for excitation and 530 nm for emission. Chlorophyll-A was detected at 488 nm for excitation and 700 nm for emission. Alternatively, the pattern of VENUS expression was also observed with a Leica CLSM model SP5 or a Zeiss lsm780 confocal microscope (VENUS excitation: 488 nm; emission: 536 nm. Chlorophyll-A excitation: 561 nm; emission: 680 nm. PI excitation: 561 nm; emission: 648 nm). VENUS expression and GUS staining were further observed with a Nikon SMZ1270 stereomicroscope equipped with a Nikon DS-RI2 camera and NIS elements software. The expression of VENUS was also quantified using ImageJ software [62].

### Tissue collection, RNA preparation and analysis

Arabidopsis RNA was extracted using the RNeasy Mini Kit (Qiagen) according to the manufacturer’s instructions, except that samples were incubated for 30 min at room temperature after addition of the lysis buffer. cDNA synthesis was performed using the SuperScript™ II Reverse Transcriptase cDNA Kit (invitrogen) with 3 µg of RNA. Tomato RNA preparation and qRT-PCR analysis were performed as previously described [63]. Quantitative reverse transcription-PCR analysis was performed using the Absolute Blue qPCR SYBR Green ROX Mix (AB-4162/B) kit (Thermo Fisher Scientific). Reactions were performed using a Rotor-Gene 6000 cycler (Corbett Research). A standard curve was obtained for each gene using dilutions of a cDNA sample. Each gene was quantified using Corbett Research Rotor-Gene software. Values are means of at least three biological repeats, each containing for tomato: the above ground tissue of 2 week-old seedlings treated as indicated (4–6 seedlings per sample), and for arabidopsis: ~ 30 µg of ten day-old seedlings treated as indicated.

Expression of tomato genes was normalized relative to tomato *EXPRESSED (EXP)*, and expression of arabidopsis genes was normalized relative to arabidopsis *TUBULIN BETA CHAIN3 (B-TUB3)*. Primer sequences used for the qRT-PCR analyses are detailed in Additional file 1: Table S1. Student’s t-test (two-tailed) was used for comparison of means, which were deemed significantly different at P ≤ 0.05.

## Supplementary information

**Additional file 1: Figure S1.** Possible dose effects of the* TCSv2*:3XVENUS response to CK treatment. Arabidopsis (top, Columbia seedlings are depicted) and tomato (bottom, M82 shoots are depicted) were treated with mock or indicated concentrations of BA (6-benzylaminopurine) for 24-48 hours. Images were taken with a Nikon stereomicroscope. Bars= 100 µM. **Figure S2.** *TCSv2* driven expression in arabidopsis in the *Ler* background. Arabidopsis seedlings in the *Ler* background expressing *TCSv2* driven VENUS (top) or GUS (bottom), with or without (mock) BA (6-benzylaminopurine) treatment, were photographed 24 hours after CK treatment or subjected to GUS staining 24 hours after CK treatment. Images were taken with a Nikon stereomicroscope. Bars= 100 µM. **Figure S3.** *TCSv2* responds primarily to CK. Characterization of *TCS *driven VENUS expression in wild type tomato shoot apexes following treatment with indicated hormones. Images were captured with a Nikon stereomicroscope. Bars= 100 µM. **Figure S4.** Stereomicroscope analysis of *TCSv2* driven expression in tomato leaves. Tomato seedlings expressing *TCSv2* driven VENUS at various stages of leaf development as indicated. Images were taken with a Nikon stereomicroscope. The VENUS channel was hue-masked using Adobe photoshop to a dark blue color in order to better visualize it when superimposed on light microscopy images of the young developing leaves. Bars= 100 µM. **Figure S5.** *TCSv2* driven expression in the tomato embryo. *TCSv2* driven expression in mature tomato embryos. Images were taken with a Nikon stereomicroscope. The root apical meristem is indicated with an asterisk (A-C) and the shoot apical meristem is marked with a dotted box (A-D). The area in the dotted box in D is enlarged in E. Bars= 100 µM. **Table S1.** Primer pairs used in this work.

## Data Availability

All data generated or analysed during this study are included in this published article and its supplementary information files.
