# Supplementary material for: Characterization of the cytokinin sensor TCSv2 in arabidopsis and tomato
Source: Plant Methods. 2020 Nov 16;16:152. doi: 10.1186/s13007-020-00694-2 (PMC7670716; doi:10.1186/s13007-020-00694-2)
Supplement: Supplementary file 1 — Additional file 1: Figure S1. Possible dose effects of the TCSv2:3XVENUS response to CK treatment. Arabidopsis (top, Columbia seedlings are depicted) and tomato (bottom, M82 shoots are depicted) were treated with mock or indicated concentrations of BA (6-benzylaminopurine) for 24-48 hours. Images were taken with a Nikon stereomicroscope. Bars= 100 µM. Figure S2. TCSv2 driven expression in arabidopsis in the Ler background. Arabidopsis seedlings in the Ler background expressing TCSv2 driven VENUS (top) or GUS (bottom), with or without (mock) BA (6-benzylaminopurine) treatment, were photographed 24 hours after CK treatment or subjected to GUS staining 24 hours after CK treatment. Images were taken with a Nikon stereomicroscope. Bars= 100 µM. Figure S3. TCSv2 responds primarily to CK. Characterization of TCS driven VENUS expression in wild type tomato shoot apexes following treatment with indicated hormones. Images were captured with a Nikon stereomicroscope. Bars= 100 µM. Figure S4. Stereomicroscope analysis of TCSv2 driven expression in tomato leaves. Tomato seedlings expressing TCSv2 driven VENUS at various stages of leaf development as indicated. Images were taken with a Nikon stereomicroscope. The VENUS channel was hue-masked using Adobe photoshop to a dark blue color in order to better visualize it when superimposed on light microscopy images of the young developing leaves. Bars= 100 µM. Figure S5. TCSv2 driven expression in the tomato embryo. TCSv2 driven expression in mature tomato embryos. Images were taken with a Nikon stereomicroscope. The root apical meristem is indicated with an asterisk (A-C) and the shoot apical meristem is marked with a dotted box (A-D). The area in the dotted box in D is enlarged in E. Bars= 100 µM. Table S1. Primer pairs used in this work. [file 13007_2020_694_MOESM1_ESM.docx]

**
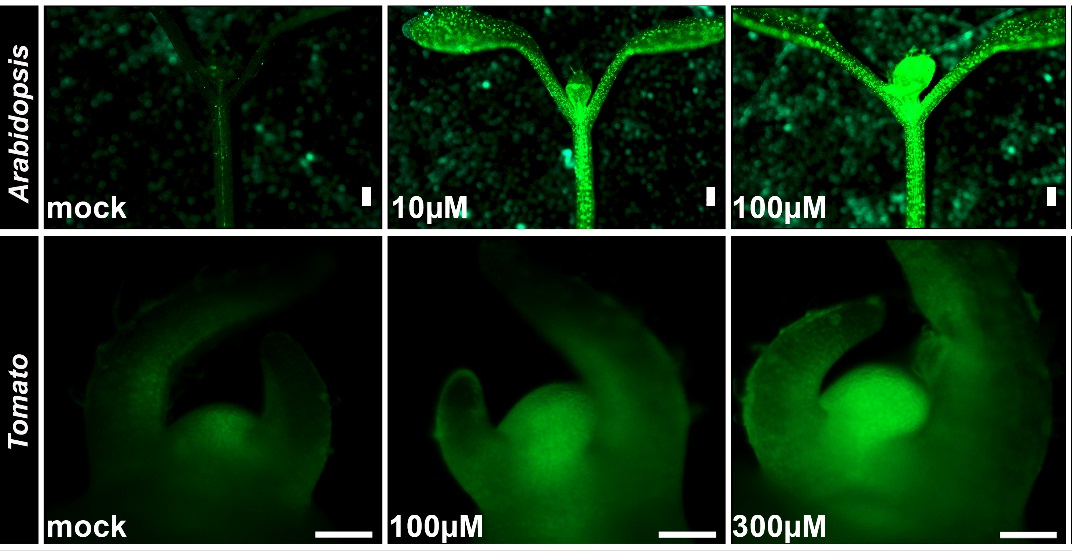
**

**Figure S1**

**Possible dose effects of the *TCSv2*:3XVENUS response to CK treatment**

Arabidopsis (top, Columbia seedlings are depicted) and tomato (bottom, M82 shoots are depicted) were treated with mock or indicated concentrations of BA (6-benzylaminopurine) for 24-48 hours. Images were taken with a Nikon stereomicroscope. Bars= 100 uM.


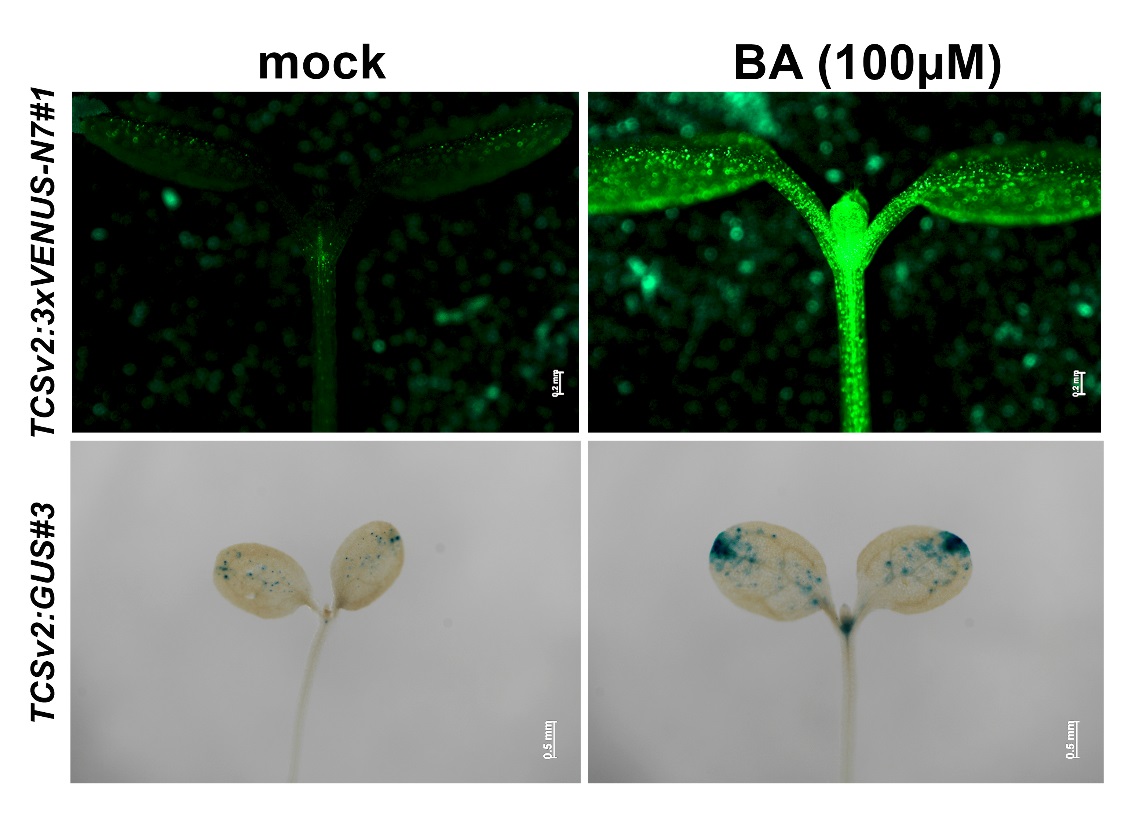


**Figure S2**

***TCSv2* driven expression in arabidopsis in the *Ler* background**

Arabidopsis seedlings in the *Ler* background expressing *TCSv2* driven VENUS (top) or GUS (bottom), with or without (mock) BA (6-benzylaminopurine) treatment, were photographed 24 hours after CK treatment or subjected to GUS staining 24 hours after CK treatment. Images were taken with a Nikon stereomicroscope. Bars= 100 uM.


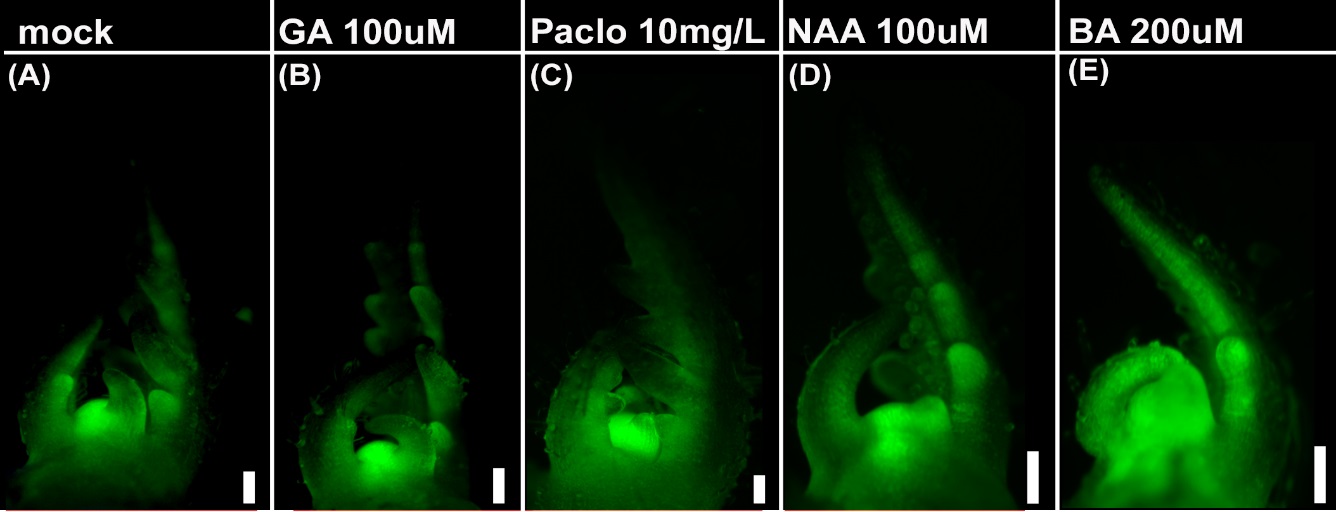


**Figure S3**

***TCSv2* responds primarily to CK**

Characterization of *TCS* driven VENUS expression in wild type tomato shoot apexes following treatment with indicated hormones. Images were captured with a Nikon stereomicroscope. Bars= 100 uM.


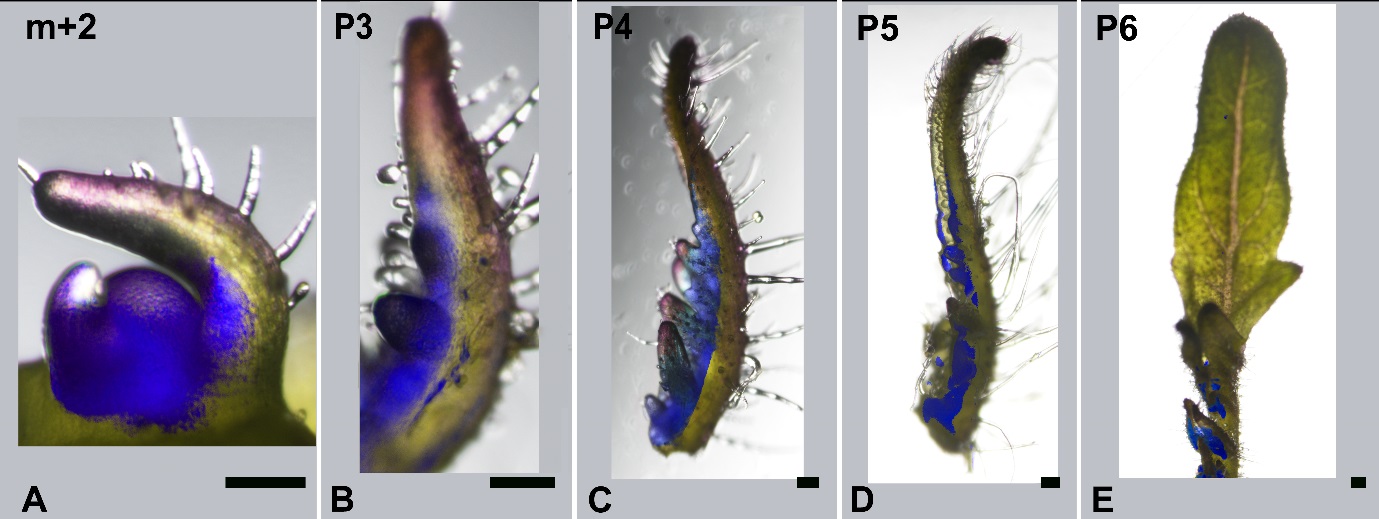


**Figure S4**

**Stereomicroscope analysis of *TCSv2* driven expression in tomato leaves**

Tomato seedlings expressing *TCSv2* driven VENUS at various stages of leaf development as indicated. Images were taken with a Nikon stereomicroscope. The VENUS channel was hue-masked using Adobe photoshop to a dark blue color in order to better visualize it when superimposed on light microscopy images of the young developing leaves. Bars= 100 uM.


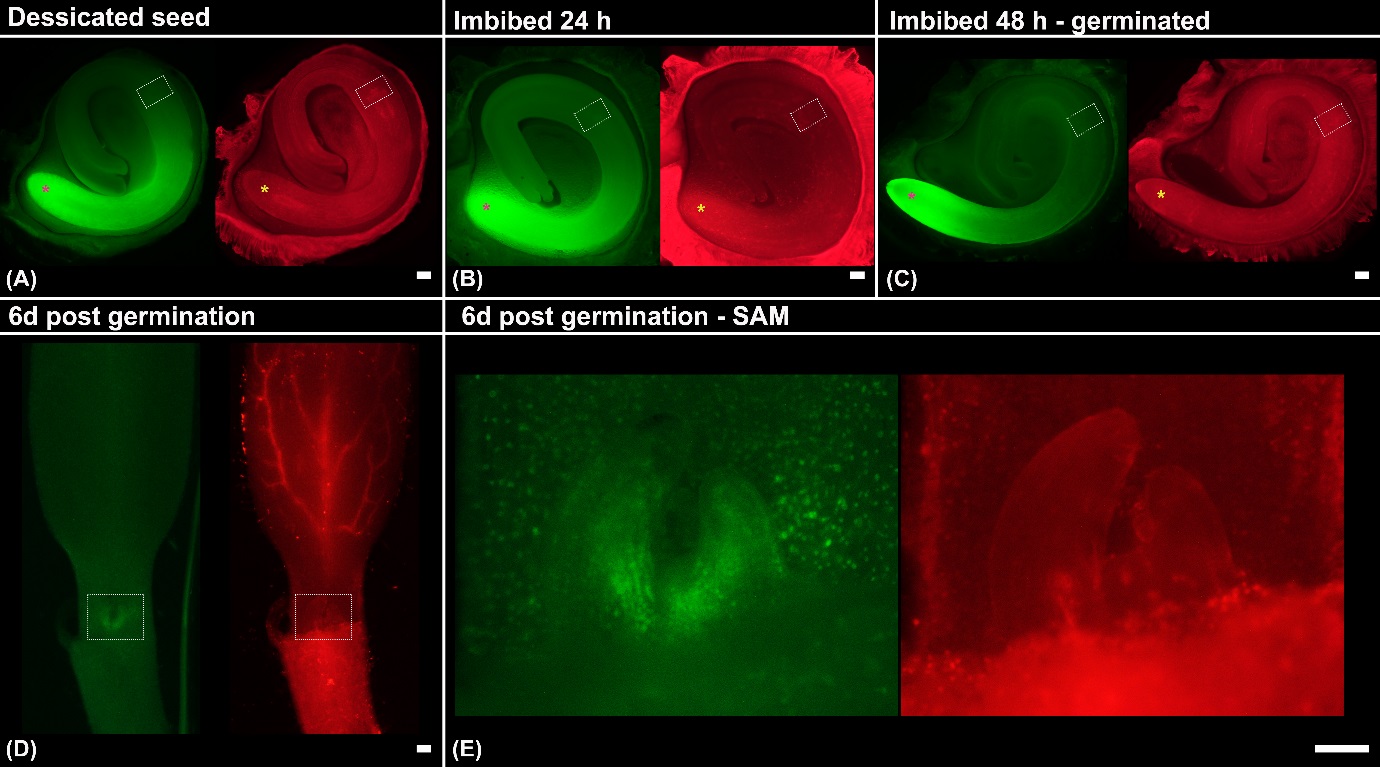


**Figure S5**

***TCSv2* driven expression in the tomato embryo**

*TCSv2* driven expression in mature tomato embryos. Images were taken with a Nikon stereomicroscope. The root apical meristem is indicated with an asterisk (A-C) and the shoot apical meristem is marked with a dotted box (A-D). The area in the dotted box in D is enlarged in E. Bars= 100 uM.

**Table S1**

**Primer pairs used in this work.**

| Name | Sequence | Use |
| --- | --- | --- |
| *EXP* RT-F | TGGGTGTGCCTTTCTGAATG | qRT-PCR tomato |
| *EXP* RT-R | GCTAAGAACGCTGGACCTAATG | qRT-PCR tomato |
| TRR3/4 F | CGTCCCCTAAAGCATTCTCA | qRT-PCR tomato |
| TRR3/4 R | CGTCTTGTTGGTGATGTTGG | qRT-PCR tomato |
| TRR5/6/7 F | GGGATTGATGGTTTGAAGGT | qRT-PCR tomato |
| TRR 5/6/7 R | ATCTTGCTCAACACCGATGA | qRT-PCR tomato |
| TRR 16b F | CATCAATGCATGGAAGAAGG | qRT-PCR tomato |
| TRR 16b R | GCATTGCATTATTTGGCATC | qRT-PCR tomato |
| B-TUB F | AAACTCACTACCCCCAGCTTT | qRT-PCR arabidopsis |
| B-TUB R | GAGAGGAGCAAAACCAACCA | qRT-PCR arabidopsis |
| ARR5 F | GAAGTTCATCGAGCGGTTACTC | qRT-PCR arabidopsis |
| ARR5 R | TTAATCTTCAGATCCTCAAATCCA | qRT-PCR arabidopsis |
| ARR7 F | CAATGCCAGGACTTTCAGGA | qRT-PCR arabidopsis |
| ARR7 R | TTTGATTCGCTTTACATCTGC | qRT-PCR arabidopsis |
| ARR16 F | TGCAAAGTGACAACAGCAGA | qRT-PCR arabidopsis |
| ARR16 R | CCAGGCATACAGTAATCGGA | qRT-PCR arabidopsis |
| GUS F | ACAGCCAAAAGCCAGACAGA | qRT-PCR tomato and arabidopsis |
| GUS R | TGACGACCAAAGCCAGTAAAG | qRT-PCR tomato and arabidopsis |
